# Evaluation of Vulnerability Status of the Infection Risk to COVID-19 Using Geographic Information Systems (GIS) and Multi-Criteria Decision Analysis (MCDA): A Case Study of Addis Ababa City, Ethiopia

**DOI:** 10.3390/ijerph19137811

**Published:** 2022-06-25

**Authors:** Hizkel Asfaw, Shankar Karuppannan, Tilahun Erduno, Hussein Almohamad, Ahmed Abdullah Al Dughairi, Motrih Al-Mutiry, Hazem Ghassan Abdo

**Affiliations:** 1 Department of Geomatics Engineering, School of Civil Engineering and Architecture, Adama Science and Technology University, Adama P.O. Box 1888, Ethiopia; hizkelasfaw353@gmail.com (H.A.); tilerduno@yahoo.com (T.E.); 2 Department of Applied Geology, School of Applied Natural Science, Adama Science and Technology University, Adama P.O. Box 1888, Ethiopia; geoshankar1984@gmail.com; 3 Department of Geography, College of Arabic Language and Social Studies, Qassim University, Burayda 51452, Saudi Arabia; adgierie@qu.edu.sa; 4 Department of Geography, Justus Liebig University of Giessen, 35390 Giessen, Germany; 5 Department of Geography, College of Arts, Princess Nourah Bint Abdulrahman University, P.O. Box 84428, Riyadh 11671, Saudi Arabia; mkalmutairy@pnu.edu.sa; 6 Geography Department, Faculty of Arts and Humanities, University of Tartous, Tartous P.O. Box 2147, Syria; hazemabdo@tartous-univ.edu.sy; 7 Geography Department, Faculty of Arts and Humanities, University of Damascus, Damascus P.O. Box 30621, Syria; 8 Geography Department, Faculty of Arts and Humanities, University of Tishreen, Lattakia P.O. Box 2237, Syria

**Keywords:** COVID-19, GIS, MCDA, proximity analysis, weighted overlay

## Abstract

COVID-19 is a disease caused by a new coronavirus called SARS-CoV-2 and is an accidental global public health threat. Because of this, WHO declared the COVID-19 outbreak a pandemic. The pandemic is spreading unprecedently in Addis Ababa, which results in extraordinary logistical and management challenges in response to the novel coronavirus in the city. Thus, management strategies and resource allocation need to be vulnerability-oriented. Though various studies have been carried out on COVID-19, only a few studies have been conducted on vulnerability from a geospatial/location-based perspective but at a wider spatial resolution. This puts the results of those studies under question while their findings are projected to the finer spatial resolution. To overcome such problems, the integration of Geographic Information Systems (GIS) and Multi-Criteria Decision Analysis (MCDA) has been developed as a framework to evaluate and map the susceptibility status of the infection risk to COVID-19. To achieve the objective of the study, data like land use, population density, and distance from roads, hospitals, bus stations, the bank, markets, COVID-19 cases, health care units, and government offices are used. The weighted overlay method was used; to evaluate and map the susceptibility status of the infection risk to COVID-19. The result revealed that out of the total study area, 32.62% (169.91 km^2^) falls under the low vulnerable category (1), and the area covering 40.9% (213.04 km^2^) under the moderate vulnerable class (2) for infection risk of COVID-19. The highly vulnerable category (3) covers an area of 25.31% (132.85 km^2^), and the remaining 1.17% (6.12 km^2^) is under an extremely high vulnerable class (4). Thus, these priority areas could address pandemic control mechanisms like disinfection regularly. Health sector professionals, local authorities, the scientific community, and the general public will benefit from the study as a tool to better understand pandemic transmission centers and identify areas where more protective measures and response actions are needed at a finer spatial resolution.

## 1. Introduction

COVID-19 is a disease caused by the SARS-CoV-2, which is a new coronavirus [1]. COVID-19 is an unintended global public health threat, especially for countries with limited resources. The disease was first identified on 31 December 2019, in Wuhan, China, and has since spread throughout the world [2]. The World Health Organization (WHO) called the COVID-19 outbreak a pandemic due to the dramatic rise in the number of cases worldwide [1]. Coronavirus disease 2019 (COVID-19) is a contagious respiratory disease caused by a new coronavirus species that cause serious clinical symptoms such as fever, dry cough, dyspnea, respiratory problems, and pneumonia. It can lead to respiratory failure and death [3,4,5]. The disease is transmitted from person to person by contaminated air droplets that are ejected through sneezing or coughing [6]. Humans who come into contact with virus-infected hands or surfaces and then touch their eyes, nose, or mouth with those hands may also spread the disease [7]. COVID-19 is a virus that affects people of all ages. However, proof to date indicates that the elderly (i.e., over 60); and those with an underlying illness (such as heart disease, diabetes, chronic respiratory disease, and cancer) are more likely to contract the serious COVID-19 disease [8]. Ethiopia is one of the most populated countries in Sub-Saharan Africa, with a population of around 115 million. It is also one of the poorest nations, with a per capita income of 850 dollars in 2019 and a human development index of 0.47, ranking 173rd out of 189 countries and territories. The country’s major health concerns are tuberculosis (TB), malaria, Human Immunodeficiency Viral Infection (HIV/AIDS), and maternal mortality, and it is one of the countries with the highest rates of TB, TB/HIV, and multidrug-resistant TB [9].

The first confirmed case of COVID-19 in Ethiopia was on 13 March 2020. As of 11 November 2020, 104,427 cases had been reported positive, with 1607 (case fatality rate, CFR = 1.5%) deaths and 64,983 (62.2%) recoveries. The primary case was a 48-year Japanese man; consequently, the second report was three cases, one Ethiopian and two Japanese, who had contact with the primary case. In the capital Addis Ababa, which is a busy center of economic, social, and political activity and home to such prominent offices as the AU, reported the first two cases of COVID-19 on 29 March 2020, both having a travel history to Dubai at different dates [10]. Before community transmission started, the cases were mostly imported and sourced from mandatory quarantines. Until 2 June 2020, 30.4 percent of all cases had been imported, indicating that the disease was contracted outside the country. The majority of the incidents, 5337 (98.4%), included Ethiopian nationals from all nine National Regional States and two City Administrations. Throughout the country, most of the cases, 3822 (71.6%), were reported from Addis Ababa [9].

Following the first case of COVID-19 in Ethiopia in March 2020, the Ethiopian government took several public health actions to halt the pandemic’s spread. On 30 March 2020, the Prime Minister called on the public to have more discipline in fighting the coronavirus’s spread in the country and appealed to the public to fulfill their responsibilities by applying all recommended prevention measures. On 8–10 April 2020, the government and parliament proclaimed a state of emergency, and on 11 April 2020, the council of ministers released a regulation. The National Ministerial Committee emphasizes, among other things, prevention and protection; a 14-day mandatory quarantine for passengers arriving in Ethiopia, avoiding public/religious assemblies; health sector capacity building; and market regulation to avoid unethical exploitation of the situation; and support for regions’ preparedness to forestall the disease [11]. Similarly, the emergency declarations and regulations stress avoiding handshakes, reducing the number of people using public transportation by half, maintaining sufficient physical distance, and providing cleaning and handwashing facilities in different parts of each city [12].

Although the measure mentioned above has been taken, the number of COVID-19 infections has proliferated. Till 1 November 2020, the growth was with an incidence rate of 650 cases per 10,000 tests at the national level. This amount has risen to 674 cases per 10,000 tests since 1 December 2020. This suggests that the rate increased by 27 more cases countrywide in only one month [13]. While in Addis Ababa, the incidence rate was 805 cases per 10,000 tests till 1 November 2020. This number escalated to 835 on 1 December 2020, increasing by 30 cases from 1 November 2020 [13]. Until 1 December 2020, total cases of 96,583 were recorded in the country and 58,853 cases in Addis Ababa. This indicated that above 50% of cases in the counties are recorded in Addis Ababa.

The above-mentioned fact implies that the pandemic is spreading at a fast rate in the city. Besides, the government has loosened restrictions on social services, religious assemblies, markets, and other suggested pandemic-prevention measures [14]. These conditions are conducive to pandemic spread and will greatly accelerate COVID-19 expansion in the upcoming months. It is then indispensable for the government and concerned bodies to have operative methodologies to help the local pandemic prevention mechanisms by strengthening decision-making in order to mitigate and control COVID-19’s spread.

Various researchers have implemented Multiple Criteria Decision Analysis (MCDA) techniques integrated with the Geographic Information System to evaluate vulnerability to infection risk and contagion [15,16,17]. These two distinctive disciplines, GIS and MCDA, can benefit from each other. According to [18], GIS has many weaknesses in spatial decision-making. Integration of GIS technology with MCDA is the solution to these limitations. Moreover, ref. [19] indicates that MCDA provides a comprehensive set of tools and approaches for organizing decision-making problems and designing, assessing, and ranking possible alternatives. Besides, ref. [20] found that GIS techniques and procedures play an important role in the analysis of decision problems. GIS is also known as a decision-making process that involves the incorporation of spatially referenced data into a problem-solving context. Therefore ref. [19] concluded as GIS-MCDA, at its most basic level, is a tool for transforming and combining geographic data with value judgments (the decision-makers preferences) to acquire information for decision making. Thus, this brings various favorable circumstances to practice for public health planning and epidemiological studies. By monitoring the source of disease and the contagion spread, agencies may respond more efficiently to disease outbreaks by identifying at-risk populations and targeting action.

Vulnerability is defined as “the conditions determined by physical, social, economic and environmental factors or processes, which increase the susceptibility of a community to the impact of hazards” [21]; it is defined in this study as the people’s susceptibility to the infection risk of COVID-19 epidemic. However, different MCDA techniques are used for vulnerability assessment; the Hierarchical Analytical Process (AHP) is one of the most commonly used approaches in decision-making processes, initially developed by [22]. The reason for its implementation is that it allows the relative value of two or more attributes to be evaluated by pairwise comparisons [23]. It is capable of taking into account all factors in a hierarchical style usually based on the perception of the person, which enables these factors to be systematically organized and their contribution to the risks to be explained with priority weights. It is commonly used since the result is simple to understand, interpret, and implement. AHP is particularly useful for addressing qualitative and quantitative multi-criteria factors in decision-making [24].

Geographical Information Systems (GIS) are now well established both as a technology and a set of tools for manipulating and displaying spatial data in an ever-increasing range of application areas [25]. Over the last few decades, the increased use of Geographic Information System technology has changed health care services and provided new insight to public health providers, researchers, hospital and health system staff, as well as the public they serve [26,27]. Though maps and spatial analysis have a long history in health and human services, the early twenty-first century is a period when health practitioners and the general public have access to a wealth of powerful spatial analytic tools [28,29]. Through vulnerability and risk analysis, spatial prediction, tracking spatial resource distributions, and providing spatial logistics for management, geographic information systems (GISs) have played a significant part in providing significant insight during previous virus outbreaks such as Zika, influenza, West Nile, Dengue, Chikungunya, Ebola, Marburg, and Nipah) [30].

This study aims to evaluate the vulnerability status of infection risk to the COVID-19 pandemic in Addis Ababa, Ethiopia, by standardizing and putting a vulnerability model into operation using Geographic Information Systems (GIS) and Multi-Decision Criteria Analysis (MCDA). Identifying vulnerable areas to infection risk of COVID-19 pandemic has an evitable role in implementing intended infection prevention and control actions to tackle the significant causative factors at their specific locations. Spatial analysis with GIS, on the other hand, can illustrate the human and ecological environment during which diseases are transmitted to spot susceptibility and possible intervention, and it can be used to detect a group of diseases and the proximity of vulnerable populations to the risk source [31]. It is anticipated that the results of this study will provide valuable scientific knowledge to decision-makers about how to use GIS tools to take steps to control and monitor pandemics and serve as a benchmark for application.

## 2. Materials and Methods

### 2.1. Description of the Study Area

**Figure 1:**
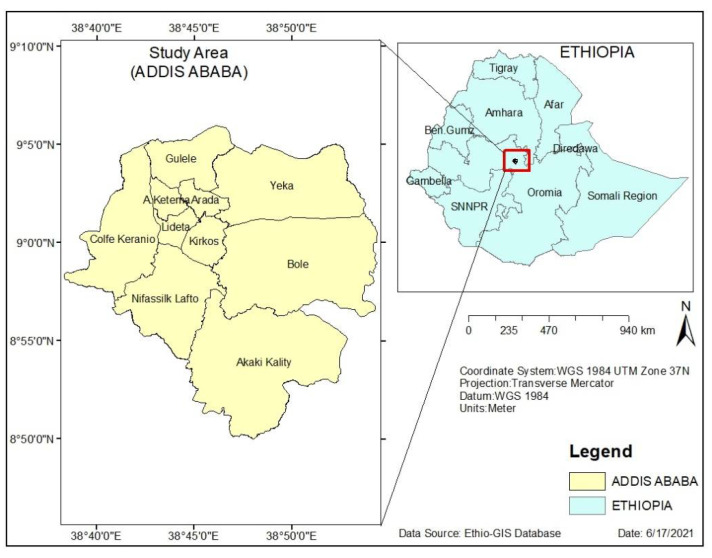
Location Map of the Study Area.

Addis Ababa is the capital city of Ethiopia and is located on latitude 90°1′48″ N and longitude 38°44′24″ E (Figure 1). The city lies at the foot of Mount Entoto with an elevation of 2355 m above sea level and covers about 540 km^2^. Addis Ababa hosts the headquarters of the African Union (AU) and the United Nations Economic Commission for Africa (UNECA). The city is divided into 10 sub-cities without newly added sub-city: Addis Ketema, Akaky Kaliti, Arada, Bole, Gullele, Kirkos, Kolfe Keranio, Lideta, Nifas Silk-Lafto, and Yeka [32]. According to 2015 estimates by the Central Statistical Agency (CSA), the total population of Ethiopia was about 90 million. Addis Ababa is home to an estimated 3,434,000 inhabitants, which is 3.6% of the national population and 18% of the total urban population of Ethiopia. The city has a population density per square kilometer of 6516 people. The population growth rate of Addis Ababa is around 2.1 percent per annum, with a projected population of 4.7 million people by 2030 [32]. Addis Ababa lies between 2200 and 2500 m above sea level. Due to its high altitude, Addis Ababa encounters a humid subtropical warm summer climate, which is mild with dry winters, mild, rainy summers, and moderate seasonality. The average annual temperature range is 10 °C to 24 °C. From February to May, the city has a short rainy season with a long wet season from June to mid-September [32].

### 2.2. Materials and Data Collection

Different data from primary and secondary sources were collected from field surveys, online sources, and respective institutions. They are summarized below in Table 1.

### 2.3. Method of Data Analysis

**Figure 2:**
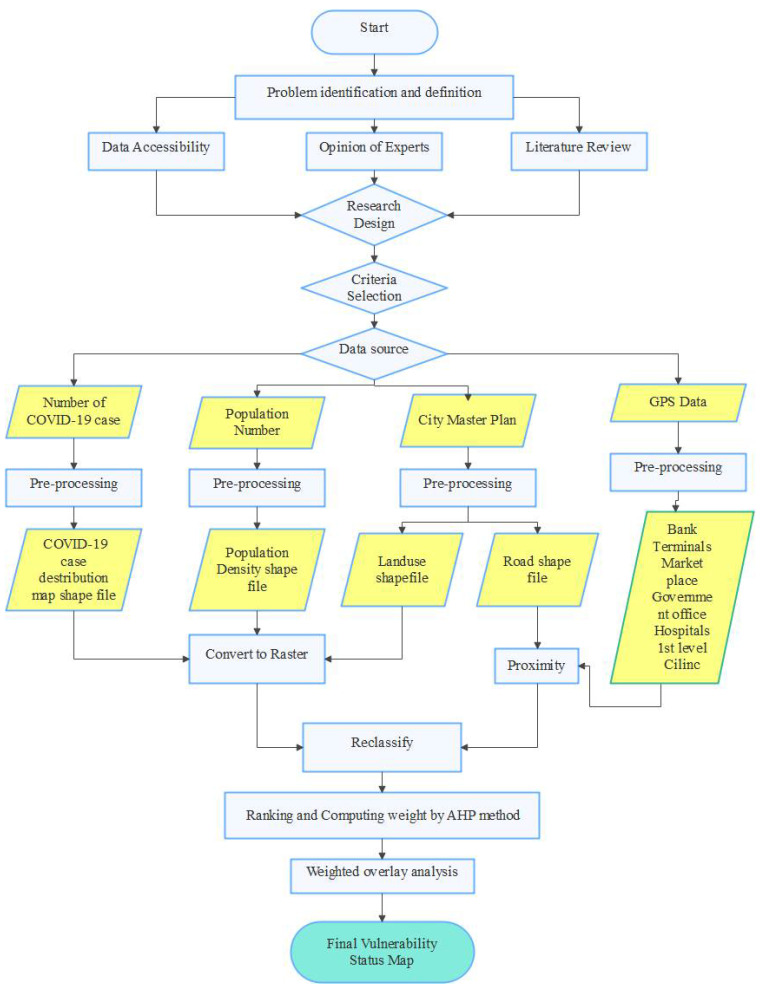
Technological scheme of the study.

After the data was collected from various sources, the data were analyzed using different software like Esri ArcGIS 10.8 and IDRISI Selva 17 (Table 2). The details of data analysis as per the conceptual framework flow chart are presented in Figure 2.

The primary step was setting the goal of defining the problem and setting the research design. In this study, GIS-based MCDA aims to produce a map showing the vulnerability status of infection risk of COVID-19 in Addis Ababa city. The following step was determining factors/criteria that are important for COVID-19 vulnerability status evaluation. It is important to choose the appropriate influential factors as their quality affects the validity of the results [33]. Since no adequate previous studies of the vulnerability status of an area to COVID-19 infection risk have been conducted, the selection of influential factors is a very challenging task. Besides, no universal factors are accepted for mapping and evaluating the vulnerability status of COVID-19 infection risk. In such circumstances, vulnerability factors can be identified by either collecting data from a literature review or prior knowledge of the phenomena. Ongoing research on pandemics has shown that local and community-wide transmission of the virus occurs mainly in public places where most people are likely to come into contact with the greatest number of possible infection carriers [34]. Evaluating the vulnerability status of infection risk of COVID-19 in Addis Ababa was selected mainly based on prior knowledge of the property of the pandemic and available literature review [15,35], which includes Landuse, Population density, distance from roads, distance from hospitals, distance from bus stations/terminals, distance from the bank, distance to Markets, number of people with COVID-19, distance to health care unite, and distance to government offices. These datasets were collected from various sources and processed using ArcGIS software for MCDA.

The third step was standardizing each factor/criterion. Data pre-processing, proximity analysis, rasterization, and reclassification are included in this step.

**Data Pre-processing:** Pre-processing activities made were 4-fold; for the number of COVID-19, population number, City Master plan, and GPS (Co-ordinate) data. The COVID-19 case distribution map was prepared by joining the numerical data of the COVID-19 case distribution with the sub-city shapefile. To pre-process the numerical data, the following systematic steps were followed. The number of COVID-19 case data was first encoded in an excel sheet with their respective sub-city and saved as CSV (comma delimited) file format. This tabular data is then joined with the attribute table of the sub-city shapefile based on a field common to both tables in ArcGIS software.

The other pre-processing activities were done for population number data. A similar approach was followed to joining the numerical data with their respective sub-city in the ArcGIS environment. Then the new population density field was added to the attribute table of the sub-city shape file next. The population density was calculated by applying Equation (1) using the field calculator tool in ArcGIS software.

Population Density = Number of People/Land Area (km^2^) (1)

The pre-processing activity for the city master plan was to extract landuse and road features (only the main road). These two factors were selected and exported separately from the city master plan by using the Export Data tool in ArcGIS software to get a shapefile of each feature. Various land-use classes in the exported land-use feature are merged into four classes that contribute to the COVID-19 spread using the Merge tool from the editor menu in ArcGIS software. Finally, road and land-use shapefile were prepared for further analysis. Finally, the GPS (coordinate) data were pre-processed. In this fold, the pre-processing activity involves the conversion of the coordinate data in excel format (CSV comma delimited) to the point feature class (shapefile). This activity was done by using the Export Data tool in ArcGIS software.

**Proximity analysis:** In order to generate the vulnerability status of COVID-19 infection risk based on nearness to the transmission source, it is necessary to apply proximity analysis (Table 3). Proximity analysis is the process of analyzing the locations of features by measuring the distance between them and other features in the area. In this study, proximity analysis was done by using Euclidean distance tools for the factors such as Road, Market Place, Bank, Hospital, 1st level Clinic, Terminal, and Government office. The ability to generate raster output from vector input without further rasterization process was the cause for using the Euclidean distance tool in this study. Euclidean distance involves determining the straight distance between a map of each cell and a source determined. It is determined from the center of a source cell to the center of each surrounding cell by locating the hypotenuse [36]. The input source data can be a feature class or raster. When the input source data is a feature class, the source locations are converted internally to a raster before performing the analysis. The resolution of the raster can be controlled with the Output cell size parameter or the Cell Size environment. In this study, 30 × 30 m cell size was assigned for all analyses. By default, the resolution will be determined by the shorter width or height of the extent of the input feature in the input spatial reference, divided by 250. When the input source data is a raster, the source cells consist of all cells in the source raster with valid values. Cells that have No Data values are not included in the source set. The value 0 is considered a legitimate source. A source raster can be easily created using extraction tools. All output raster in Euclidean distance is of floating-point type.

**Rasterization:** To make data suitable for reclassification and further analysis, vector data must be rasterized. Simply rasterization is a process of converting vector (feature-based) data into raster (pixel-based) format. This study used a Conversion tool, specifically, the polygon to raster tool in the ArcGIS environment for the factors of Landuse, Population density, and COVID-19 distribution map. 30 × 30 m cell size was assigned for all raster layers.

**Reclassification:** Data depicted on an ongoing scale are the resulting maps generated from the Euclidean analysis and Rasterization. Each range must be assigned a single value before it can be used in the weighted overlay tool to make comparisons possible. The Reclassify tool allows for such a raster to be reclassified. Therefore, in this study, the ranges of values generated in the Euclidean analysis and rasterization were reclassified into 4 groups, using equivalent interval methods in ArcGIS software. A general score ranging from 1 to 4 was allocated to the groups. A score of 1 is a low vulnerability, while a score of 4 is extremely high vulnerability.

The fourth step was defining weights for each criterion based on its influence on the vulnerability of infection risk of COVID-19. Several methods are available to determine the weight, like Analytical Hierarchy Process (AHP), ranking, and rating. In this study, the AHP was used to assess each selected factor’s importance. AHP uses a hierarchical structure; it enables decision-makers to define high-level strategic objectives and specific metrics to evaluate strategic alignment better. It can be applied in any organization with any level of maturity because the inputs are normalized using either numerical data or subjective judgments when metrics are not available, and the process gives itself to sensitivity analysis [22]. In the AHP, all possible combinations of two factors were compared based on expert judgment and consensus on each factor’s weight. Then a pairwise comparison matrix was prepared by assessing the factor’s importance relative to the importance of the other factor in the pair. As shown in Table 4, the importance of each factor relative to the other in a pair would have a value ranging from 1/9 (extremely less important) to 9 (extremely more important). The resulting matrix was used as an input to calculate a set of weights and consistency ratios (Table 5). The factor weighting process was done by using the IDRISI Selva Software.

**Figure 3:**
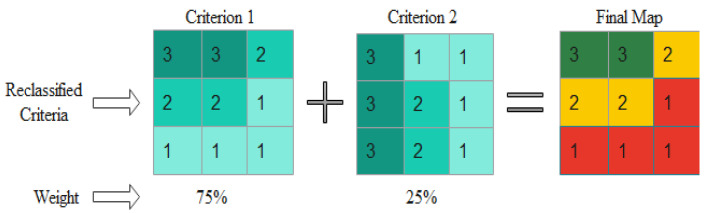
A schematic example of the final map generation weight overlay method.

Step five was to aggregate the criteria using weighted overlay analysis. Validating the result was the final step. In overlay analysis, different layers were overlaid together with their weights to generate a vulnerability status map using the weighted overlay tool. The weights assigned to the criteria represent the strength of influence of each criterion within the scenario. The values of each pixel from each criterion are multiplied by its corresponding weight (Figure 3). This is repeated for each criterion; the resulting values are added and then rounded to the nearest integer before assigning them to each pixel to obtain a final vulnerability status map. The lower the cumulative value for each pixel within the scenario, the lower the vulnerability. Conversely, the higher the cumulative value for each pixel, the higher the vulnerability.

## 3. Results and Discussion

### 3.1. Factors for COVID-19 Infection Risk Vulnerability Status Evaluation

**Figure 4:**
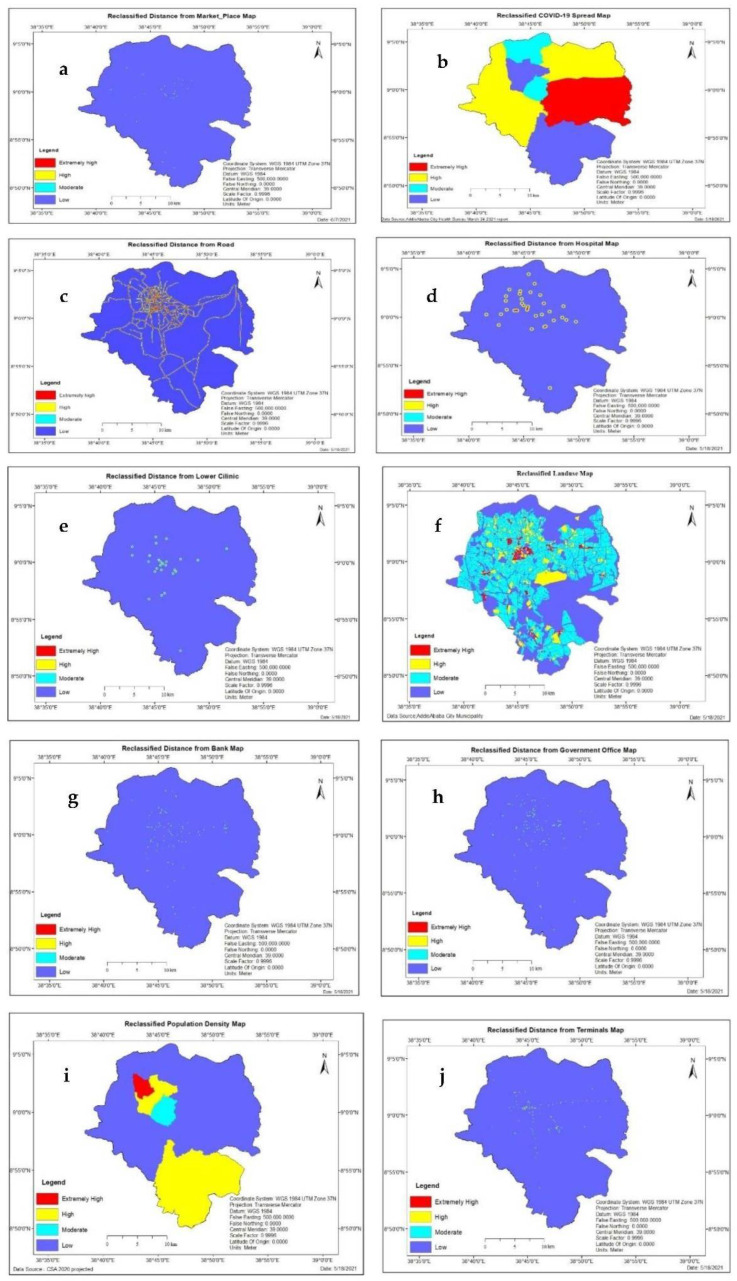
Standardized factors: Market place (**a**); COVID-19 case till 24 March 2021 Terminals (**b**); d-Road (**c**); Hospitals (**d**); Lower Clinics (**e**); Land use (**f**); Banks (**g**); Government office (**h**); Population density (**i**); Terminals (**j**).

*Proximity to Marketplace*: Markets are a critical place of commerce and a source of many essential goods, but they can pose potential risks for the COVID-19 spread [37]. In this study, the analysis results show that 0.07 km^2^ was found to be extremely high vulnerable, 0.25 km^2^ of the study area is highly vulnerable, 0.4 km^2^ was observed as moderately vulnerable area, and the rest of 520.20 km^2^ of the area is less vulnerable to infection risk of COVID-19 pandemic in terms of proximity to the marketplace. As shown in Figure 4a, an extent of 0.01% of the area was extremely high vulnerable to the infection risk of COVID-19. Since the area is the closest to the market and is highly impacted by the cases in the market due to human-to-human or human-to-object contact, it will be analyzed. Moreover, 0.05% and 0.08% of the total areas were highly and moderately vulnerable, respectively. The rest, 99.87% of the area, was low vulnerable to infection risk of COVID-19 pandemic due to its farness to transmission center (see Table 6).

*Proximity to Terminals:* The analysis results show that 0.46 km^2^ was found to be extremely high vulnerable, 0.68 km^2^ is highly vulnerable, 1.17 km^2^ was observed as a moderately vulnerable area, and the rest of 518.6 km^2^ of the area is less vulnerable for infection risk of COVID-19 pandemic. Figure 4j shows that 99.56% of the study area is low vulnerable. 0.09% is extremely high vulnerable to infection risk of COVID-19 in terms of proximity to terminals. Also, 0.13% and 0.22% of the study area are high and moderately vulnerable, respectively (see Table 6).

*COVID-19 positive case:* The results show that 119.66 km^2^ was extremely high vulnerable to infection risk of COVID-19 out of the total study area. The high vulnerable area shared 203.85 km^2^. The moderate vulnerable area accounted for 45.84 km^2^. The rest area, which is 151.56 km^2^, was evaluated as low vulnerable to infection risk of COVID-19. As shown in Figure 4b, 29.1% of the study area is less vulnerable. 22.97% of the total study area is extremely high vulnerable to infection risk of COVID-19 from the number of COVID-19 positive case points. Also, 39.13% and 8.8% of the study area are high and moderately vulnerable, respectively (see Table 6).

*Proximity to Road:* The results show that 9.1 km^2^ was extremely high vulnerable to infection risk of COVID-19 out of the total study area. The high vulnerable area shared 13.35 km^2^. The moderate vulnerable area accounted for 17.14 km^2^. The rest area, which is 481.32 km^2^, was evaluated as low vulnerable to infection risk of COVID-19. This means that 92.4% of the study area is low vulnerable, 1.75% of the total study area is extremely high vulnerable to infection risk of COVID-19 in terms of proximity to the road, and 2.56% and 3.29% of the study area are high and moderately vulnerable respectively (see Table 6).

*Proximity to Hospitals:* The results show that 2.56 km^2^ was extremely high vulnerable to infection risk of COVID-19 out of the total study area. The high vulnerable area shared 6.79 km^2^. The moderate vulnerable area covers 2.91 km^2^ of an area. The rest area, which is 508.68 km^2^, was evaluated as low vulnerable to infection risk of COVID-19. This indicated that 97.65% of the study area is less vulnerable. 0.49% of the total study area is extremely high vulnerable to infection risk of COVID-19 in terms of proximity to hospitals. Also, 1.3% and 0.56% of the study area are high and moderately vulnerable, respectively (see Table 6).

*Proximity to Lower Clinics:* In this study, the analysis results show that 0.48 km^2^ was found to be extremely high vulnerable, 1.46 km^2^ of the study area was highly vulnerable, 2.14 km^2^ was observed as a moderately vulnerable area, and the rest of 516.86 km^2^ of the area which is less vulnerable for infection risk of COVID-19 pandemic in terms of proximity to lower clinics. Figure 4e shows that 99.22% of the study area is low vulnerable. 0.09% of the total study area is extremely high vulnerable to infection risk of COVID-19 in terms of proximity to lower clinics. Also, 0.28% and 0.41% of the study area are high and moderately vulnerable, respectively (see Table 6).

*Proximity to Banks:* In this study, the results reveal that 0.43 km^2^ was found to be extremely high vulnerable, 0.67 km^2^ of the study area was highly vulnerable, 1.19 km^2^ was observed as a moderately vulnerable area, and the rest of 518.62 km^2^ of the area is less vulnerable for infection risk of COVID-19 pandemic. Figure 4g shows that 99.56% of the study area is less vulnerable. On the other hand, 0.08% of the total study area is exceptionally high vulnerable to infection risk of COVID-19 from the number of COVID-19 positive case points. Also, 0.13% and 0.23% of the study area are high and moderately vulnerable, respectively, in proximity to the bank (see Table 6).

*Proximity to Government office:* In this study, the analysis results show that 0.29 km^2^ was found to be extremely high vulnerable, 0.9 km^2^ of the study area is highly vulnerable, 1.37 km^2^ was observed as a moderately vulnerable area, and the rest of 518.35 km^2^ of the area is less vulnerable to infection risk of COVID-19 pandemic. Figure 4h shows that 99.51% of the study area is less vulnerable. On the other hand, 0.06% of the total study area is extremely high vulnerable to infection risk of COVID-19 from the number of COVID-19 positive case points. Also, 0.17% and 0.26% of the study area are high and moderately vulnerable, respectively, in proximity to a government office (see Table 6).

*Population density:* In this study, the results show that 8.63 km^2^ was found to be extremely high vulnerable,142.93 km^2^ of the study area is highly vulnerable, 14.65 km^2^ was observed as a moderately vulnerable area, and the rest 354.69 km^2^ of the area is less vulnerable for infection risk of COVID-19 pandemic. Figure 4i shows that an extent of 1.66% of the area was extremely high vulnerable to infection risk of COVID-19 due to the dense population; hence, urban sectors with high population density are more vulnerable to the spread of contagious diseases. This is due to space limitations within and between households, high mobility, and limited water, sanitation, and hygiene infrastructure. 27.44% and 2.81% of the total areas were highly and moderately vulnerable, respectively. Moreover, 68.09% of the study area was less vulnerable due to its dispersed population; hence, it’s less vulnerable to COVID-19 transmission (see Table 6).

*Landuse:* In this study, all landuse classes in the city were categorized into four. Commerce, mixed residence, service (Administrative service, municipal service, social service, infrastructure service, transport, and street network), and Miscellaneous (Environmental protection, manufacturing and storage, special projects, sports fields, urban agriculture, and historical conservation site). The result has shown that an extent of 1.44% of the area was extremely high vulnerable to infection risk of COVID-19. The area with extremely high vulnerability status to infection risk of COVID-19 is a commercial area since urban sectors with commercial land use classes are more exposed to the spread of contagious diseases. This is due to space limitations within an area, high mobility, and a high degree of people and people’s access to object contact. 7.68% and 42.38% of the total areas were highly and moderately vulnerable, respectively, and had mixed residence, service, and land use, classes. Finally, 48.50% of the study area with miscellaneous land use classes was less vulnerable to the infection risk of COVID-19. The analysis results show that 7.48 km^2^ was found to be extremely high vulnerable, 40.03 km^2^ of the study area is highly vulnerable, 220.78 km^2^ was observed as a moderately vulnerable area, and the rest 252.69 km^2^ of the area is low vulnerable for infection risk of COVID-19 pandemic in terms of land use type (see Table 6).

### 3.2. Overlaying and COVID-19 Infection Risk Vulnerability Status Evaluation

**Figure 5:**
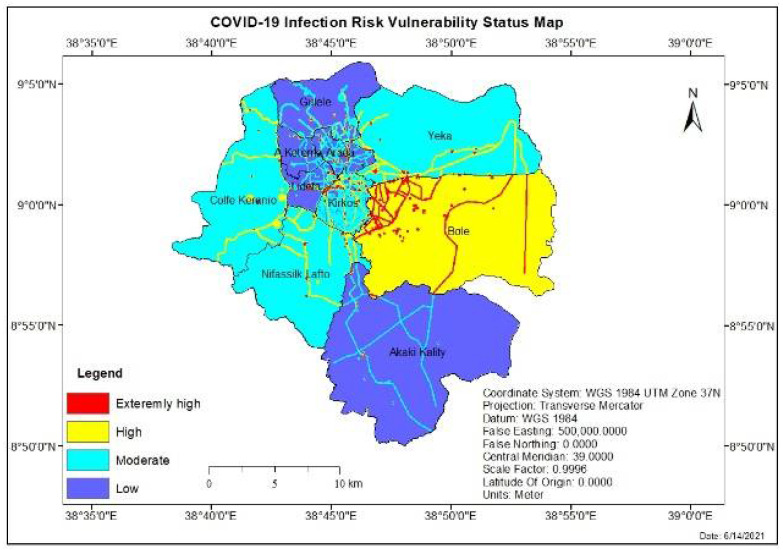
COVID-19 Infection risk vulnerability status map in Addis Ababa.

Using the weight of Factors from the pair-wise comparison eigenvectors and the reclassified factors layer as an input, four areas of COVID-19 infection risk vulnerability were obtained for weighted overlay analysis in ArcGIS, as shown in Figure 5. The result showed that out of the total study area, 32.62% (169.91 km^2^) fall under Low vulnerable (1), and the area which covers 40.9% (213.04 km^2^) is under moderate vulnerable class (2) for infection risk of COVID-19. The highly vulnerable (3) covers an area of 25.31% (132.87 km^2^), and the remaining 1.17% (6.12 km^2^) is under an extremely high vulnerable class (4). The result allowed defining areas with extremely high vulnerability; most areas are located in the central part of the city, such as Bole and Megenagna, where most of the establishments for public use are located. However, due to the conditions of the criteria, it is possible to observe large areas scattered throughout the city, covering different urban sectors. The areas are characterized by high vehicle-pedestrian traffic. The areas with high vulnerability are delimited mostly in the Bole sub-city. Hence, the area is characterized by a high number of COVID-19 confirmed cases in the city, according to the city health bureau’s report. The area with high vulnerability is also delimited by the main road around most parts of the city. The area of moderate vulnerability is located in the western, northeastern, and partially central parts of the city without a defined pattern. Finally, low vulnerability zones presented a more stable pattern in the northern and southern parts of the city. Despite having the highest surface percentage, not being close to or immersed in points of high confluence means that these areas are not risky and can be passable by applying prevention measures and security established by local governments. Hence, the maps were constructed to allow targeting intervention within the city according to the vulnerability gradient. Thus, these priority zones could regularly address pandemic control mechanisms like disinfection.

## 4. Conclusions

The study evaluates the vulnerability status of infection risk to COVID-19 by integrating Geographic Information Systems and Multi-Criteria Decision Analysis for Addis Ababa City. In this study, GIS-MCDA-based COVID-19 infection risk vulnerability status evaluation method is applied. This research has revealed that GIS integrated with MCDA is important to create operational maps that could help the health officials and all concerned identify vulnerable and priority areas for COVID-19 pandemic control. This can be considered a significant step forward for the health sector in applying geospatial technologies to support pandemic control strategies and decision-making in Ethiopia. This research can substantially contribute to the health sector for the smooth and effective implementation of adopted epidemic prevention and control strategies. Since the determination of infection risk vulnerability requires exploiting Social, Epidemiology, and GIS, this multidisciplinary knowledge requires the cooperation of various experts. The result of this study is consistent with the argument that the place with higher mobility and interaction has a high tendency of being a COVID-19 infection risk vulnerable area. The study indicated that a higher mobility and crowdedness results in a higher probability of COVID-19 spread and hence a higher COVID-19 infection risk vulnerability status. Vulnerability in a broad context is a complex process that requires understanding different input factors. However, the previous studies were based on only a few infection risk vulnerability factors as this research incorporates various infection risk vulnerability input factors. Although the success was demonstrated due to the inadequacy of data in terms of quality and quantity, the full potential of the GIS-MCDA has not been proven. Thus, further improvements are expected to result in an improved understanding of the infection risk vulnerability status by considering much more comprehensive criteria and accurate data.

## 5. Recommendations

The findings of this particular study have shown that GIS-MCDA is a useful tool for infection risk vulnerability status evaluation studies. The following are also recommended as potential research recommendations, based on the results of the study:

- One of the results noticed in this study was that an area with a cluster of services and high mobility is at a status of extremely high vulnerability to infection risk of COVID-19. Therefore, proper strategies have to be implemented to halt the spread of the disease with special attention in those areas.
- The city should develop the capacity to use GIS and MCDA to identify the vulnerability status of infection risk of COVID-19 and related activity in the city.
- It will be efficient if the city will incorporate the infection risk vulnerability status maps, which are the results of this study, in the current ongoing pandemic control activities to target the priority areas identified by the study;
- The establishment of health centers and other pandemic control facilities should be made in high and extremely high-vulnerable areas at a reasonable distance away from the existing facilities so that people at a distance can easily access the service.
- Proper databases about detailed patient data, the spatial spread of epidemics, and other related aspects of the pandemic should be made using GIS.

## Figures and Tables

**Table 1:** Data Source.

S.No	Data	Source	Data Type
1	Addis Ababa City boundary	Urban planning office	Shapefile
2	Coordinate data	Google Earth and filed work	CSV
3	Population data	Central Statistical Agency (CSA) 2020 Projected.	Text
4	Addis Ababa City Master Plan	Addis Ababa City Municipality	Shapefile/CAD file

**Table 2:** Instruments & Materials Used.

No.	Material/Software Name	Purpose
1	Handheld GPS eTrex 10	To collect the coordinate of a point
2	Esri ArcGIS 10.8	For data analysis and mapping; For Editing spatial data in arc Map and non-spatial data in arc catalog.
3	IDRISI Selva 17	To calculate the weight of each layer.
4	Google Earth Pro	To visualize spatial features in the study area and get coordinates points.
5	MS-Excel and MS-Word 2019	Integrating attribute data and preparing a thesis report

**Table 3:** Factor’s classification parameters.

Rank	Criteria	Proximity Distance (m) and Classification Parameters Extremely High to Low Vulnerable	Percent
1	Market place	>30, 30–60, 60–90, 90<	31
2	Terminals	>30, 30–60, 60–90, 90<	19
3	COVID-19 case till 24 March 2021	14,850–28,735, 10,769–14,850, 8145–10,769, 6874–8145	18
4	Road	>30, 30–60, 60–90, 90<	9
5	Government office	>30, 30–60, 60–90, 90<	6
6	Hospitals	>30, 30–60, 60–90, 90<	5
7	Lower Clinics	>30, 30–60, 60–90, 90<	4
8	Banks	>30, 30–60, 60–90, 90<	3
9	Population density	20,287–39,801, 11,456–20,287, 5615–11,456, 3479–5615	3
10	Land use	Commerce, Mixed residence, Service, Miscellaneous	2

**Table 4:** A Nine-point continuous comparison scale.

Less Important				More Important				
Extremely	Very Strong	Strong	Moderately	Equal	Moderately	Strong	Very Strong	Extremely
1/9	1/7	1/5	1/3	1	3	5	7	9

**Table 5:** Factors and their eigenvectors weights.

Factors	Mp	T	C	R	Go	H	LC	B	Pd	LU	Weight	% of Weight
Market place (Mp)	1										0.3100	31
Terminals (T)	1/3	1									0.1938	19
COVID-19 case (C)	1/3	1/2	1								0.1748	18
Road (R)	1/5	1/3	1/3	1							0.0872	9
Government office (Go)	1/5	1/3	1/5	1/2	1						0.0643	6
Hospitals (H)	1/7	1/5	1/5	1/3	1/2	1					0.0509	5
Lower Clinics (LC)	1/7	1/5	1/5	1/3	1/3	1/2	1				0.0436	4
Banks (B)	1/7	1/7	1/5	1/3	1/2	1/3	1/3	1			0.0311	3
Population density (Pd)	1/7	1/7	1/7	1/3	1/3	1/2	1/2	1/2	1		0.0267	3
Landuse (LU)	1/7	1/7	1/7	1/5	1/3	1/5	1/5	1/3	1/3	1	0.0177	2
Total											1	100

Consistency ratio = 0.06 < 0.1 = Consistency is acceptable.

**Table 6:** Reclassified parameters and area coverage of vulnerability status.

No	Factors	Proximity to Market Place (m)	Vulnerability Status	Area Coverage (km^2^)	% of Area Coverage	Rank
1.	Distance from marketplace	>90	Low	520.20	99.87	1
		60–90	Moderate	0.4	0.08	2
		30–60	High	0.25	0.05	3
		<30	Extremely high	0.07	0.01	4
2.	Distance from terminals	>90	Low	518.6	99.56	1
		60–90	Moderate	1.17	0.22	2
		30–60	High	0.68	0.13	3
		<30	Extremely high	0.46	0.09	4
3.	COVID-19 spread	6874–8145	Low	151.56	29.1	1
		8145–10,769	Moderate	45.84	8.8	2
		10,769–14,850	High	203.85	39.13	3
		14,850–28,735	Extremely high	119.66	22.97	4
4.	Distance from road	>90	Low	481.32	92.4	1
		60–90	Moderate	17.14	3.29	2
		30–60	High	13.35	2.56	3
		<30	Extremely high	9.1	1.75	4
5.	Distance from hospitals	>90	Low	508.68	97.65	1
		60–90	Moderate	2.91	0.56	2
		30–60	High	6.79	1.3	3
		<30	Extremely high	2.56	0.49	4
6.	Distance from lower clinics	>90	Low	516.86	99.22	1
		60–90	Moderate	2.14	0.41	2
		30–60	High	1.46	0.28	3
		<30	Extremely high	0.48	0.09	4
7.	Distance from banks	>90	Low	518.62	99.56	1
		60–90	Moderate	1.19	0.23	2
		30–60	High	0.67	0.13	3
		<30	Extremely high	0.43	0.08	4
8.	Distance from government office	>90	Low	518.35	99.51	1
		60–90	Moderate	1.37	0.26	2
		30–60	High	0.9	0.17	3
		<30	Extremely high	0.29	0.06	4
9.	Population density	3479–5615	Low	354.69	68.09	1
		5615–11,456	Moderate	14.65	2.81	2
		11,456–20,287	High	142.93	27.44	3
		20,287–39,801	Extremely high	8.63	1.66	4
10.	Land use	Miscellaneous	Low	252.69	48.50	1
		Service	Moderate	220.78	42.38	2
		Mixed residence	High	40.03	7.68	3
		Commerce	Extremely high	7.48	1.44	4

## Data Availability

The authors declare no competing interests. The datasets used and/or analyzed during the current study are available in the article/from the corresponding author on request.
